# Subjective Happiness Among Polish and Hadza People

**DOI:** 10.3389/fpsyg.2020.01173

**Published:** 2020-06-09

**Authors:** Tomasz Frackowiak, Anna Oleszkiewicz, Marina Butovskaya, Agata Groyecka, Maciej Karwowski, Marta Kowal, Piotr Sorokowski

**Affiliations:** ^1^Institute of Psychology, University of Wrocław, Wrocław, Poland; ^2^Smell and Taste Clinic, Department of Otorhinolaryngology, Carl Gustav Carus Medical School, TU Dresden, Dresden, Germany; ^3^Institute of Ethnology and Anthropology, Russian Academy of Sciences, Moscow, Russia; ^4^Center for Social Anthropology, Russian State University for the Humanities, Moscow, Russia

**Keywords:** positive psychology, happiness, subjective happiness scale, life satisfaction, Hadza, modernization

## Abstract

Life satisfaction and happiness were broadly studied in Western populations, whereas evidence from traditional societies remains surprisingly scarce. We collected data on the happiness from 145 Hadza, and compared it with data obtained from 156 Poles, representing Westernized society. Participants were asked to answer four simple questions from Subjective Happiness Scale (Lyubomirsky and Lepper, 1999). Results indicate that Hadza report a higher level of happiness with their lives than do Polish people. Our findings also show that sex was not related to happiness in both populations, while age was a negative predictor of happiness, but only among Poles. Therefore, we hypothesize that positive perception of aging in societies may increase their actual happiness.

## Introduction

Life satisfaction and happiness in developed, Western countries are continuously monitored by means of complex socio-economical indices. Numerous studies have been conducted on large scale samples (Eurofound, 2017), or even on samples representative for the humanity (Gallup Organization, 2016). Existing research has also focused on happiness and well-being of people from developing countries from Africa, Asia or South America (Chindarkar et al., 2018; Goodman et al., 2018; Markussen et al., 2018; Tran et al., 2018; Deng et al., 2019). However, according to our knowledge, data on the happiness in indigenous, traditional societies is remarkably scarce.

Are traditional societies happy? Gallup World Poll (see Diener et al., 2018) study including samples from 166 countries has shown that the level of happiness and life satisfaction are functions of social and material quality of life. Other studies found that objective measurements, such as infrastructure facilities, can significantly raise the subjective well-being and happiness of the people (Devoto et al., 2012; Mahasuweerachai and Pangjai, 2018). Knowing that material resources are an important predictor of happiness and life satisfaction, one could expect that traditional life in tough conditions should decrease happiness.

However, at least three studies suggested that traditional societies have relatively high levels of well-being. Evidence from the field studies suggests that Inuit, Maasai or Amish report generally high levels of happiness and life satisfaction (Biswas-Diener et al., 2005), with the most traditional Massai group showing the highest levels of general life satisfaction. Also, Himba – remote herding people of north-west Namibia inhabiting rural areas – had significantly higher levels of life satisfaction compared to the urban Himba living in towns; moreover, both Himba populations had significantly higher life satisfaction than the sample of United Kingdom adults (Martin and Cooper, 2017). Additionally, studies among Tsimane Amerindian from Amazonia suggest that, for a society in the early stages of integration to the market economy, consumption of market goods is not associated with frequency of smiles (Masferrer-Dodas et al., 2012). Altogether, these results suggest that modernization of traditional societies does not have a direct, straightforward impact on people’s happiness (see also Godoy et al., 2009, 2010), and one could also argue that modernization may even be associated with certain disadvantages.

The studies on how the quality of life and happiness of traditional people change in the course of life and between the generations are very scarce. Particularly, evidence on the perception of elderly people’s happiness is missing. Perception of aging is a socio-biological, multidimensional process varying between modern (Giles et al., 2003; Löckenhoff et al., 2009), and traditional cultures (Sorokowski et al., 2017b). Usually the concept of an old person brings together positive and negative attributions, with the latter contributing more to the general, automatic stereotype of *ageism* (Barrow and Smith, 1979; Perdue and Gurtman, 1990; Cuddy et al., 2005; Hooyman and Kiyak, 2008). Entering an old age and related decrease in psychophysical functions (Doty et al., 1984; Geffen et al., 1990; Fahle and Daum, 1997; Rantanen et al., 1999), and economic situation (Walker, 1980, 1981; Barrientos et al., 2003; Gasparini et al., 2009) diminishes the position of old people in the social exchange processes. In the modern economy experience, knowledge, wisdom, and care that old people can offer to the younger generations can be replaced or bought (e.g., information can be sought on the Internet, care services can be bought). On the contrary, in traditional societies old people might carry and transmit traditional knowledge, wisdom or experience (e.g., MacDonald, 2007). Their high competence may elicit more favorable attitudes, based on respect and esteem (Simmons, 1946; Werner, 1981; Branco and Williamson, 1982; Palmore and Maeda, 1985; Ishii-Kuntz, 1997; Eyetsemitan, 2002; Sorokowski et al., 2017b). Since positive perception of aging in societies may increase their actual quality of life and longevity (Levy et al., 2002, 2014; Ingrand et al., 2018), we can predict that happiness will increase as a function of age in traditional societies, which, in turn, would not be observed in Western societies.

To date, the level of happiness among hunter-gatherer societies has never been measured. Lifestyle in traditional societies vary significantly and this may have implications for the burden associated with taking care of older adults, who experience health problems and functional limitations. For instance, in sedentary and agricultural communities, it might be easier to care for older adults than in nomadic societies who constantly move. Moreover, in sedentary and agricultural communities, elderly people may present a higher socio-economic status and power (Sorokowski et al., 2017b) due to the higher amount of accumulated goods over the lifetime, and thus, present a higher level of happiness than hunter-gatherers, who are known to be egalitarian and do not accumulate goods (Woodburn, 1982; Marlowe, 2010; Butovskaya, 2013; Blurton Jones, 2016). Therefore, high levels of happiness and life satisfaction in elderly Hadza should not be attributed to their cumulative material status, but rather to their respectable role in the group. Furthermore, it has been previously shown that there are less social comparisons in egalitarian societies (Woodburn, 1982), as the distribution of goods is rather fair among the members of the group (Marlowe, 2004, 2009; Apicella et al., 2012; Henrich, 2012). Thus, individuals from Hadza society may not perceive huge discrepancies between them and others, in terms of acquired wealth. On the contrary, individuals from Western societies may always find numerous people who present a higher standard of living, which can lead to, for instance, envy (Guignard, 2018), and decreased happiness (Clark et al., 2006; Ball and Chernova, 2008).

Exploring the levels of happiness among hunter-gatherers may have another, practical implication. By investigating members of hunter-gatherer societies, leading the most traditional way of living and social organization, to some extent, we can address the question if global modernization and industrialization made us happier as compared to our ancestors? To this end, we collected data on the happiness of members of the Hadza society (Tanzania) (Marlowe, 2002, 2010; Butovskaya, 2013; Blurton Jones, 2016; Woodburn,2017a, b), representing one of the last traditional hunting and gathering way of living (Henrich, 2012), and compared them with Polish sample representing modern society.

## Study 1

Prior to the main, second study, we have conducted the first, pilot study, aim of which was to ensure a task comprehension among Hadza, and gather potential qualitative information regarding the applied methods. Although the first study is not an exact pilot as we have used an extended scale in the second study), in accordance with open science practices, we report its findings.

### Materials and Methods

#### Participants

Power analysis revealed that in order to obtain 95% of power within linear regression with an alpha level set to 5% and expected small-to-medium effect size of *f* = 0.1 (Löckenhoff et al., 2009; Chan et al., 2012), at least 191 participants were needed. Ninety-six Hadza (47 females, *M*_age_ = 37.8; *SD* = 15.1; Md = 36.5, Min = 17; Max = 75) were recruited for the study, and 124 Polish participants constituted as a comparison sample (66 females, *M*_age_ = 36; *SD* = 13.4; Md = 35, Min = 18; Max = 76 matched in terms of sex, χ^2^(1) = 0.39, *p* = 0.53, and age, *t*(218) = 0.93, *p* = 0.35). We deliberately overshoot the projected sample size because of expected missing data. Data from three Hadza was discarded due to the low comprehension of the task.

Because of their uniqueness, Hadza society has been extensively studied and then described in the literature. The Hadza are traditional hunter-gatherers (Marlowe, 2010; Blurton Jones, 2016). Their population has shrunk to approximately 1200 people (Blurton Jones et al., 1992; Blurton Jones, 2016). Part of the Hadza society continue the semi-nomadic way of life, establishing temporary camps, and changing locations every 1–3 months in savannah-woodland around Lake Eyasi in Northern Tanzania. According to Marlowe (2010), despite the Hadza have had contacts with non-foragers for many decades or even for centuries, they have change little. Hadza are considered to be egalitarian, with no clear hierarchy (Woodburn, 1982; Marlowe, 2010; Butovskaya, 2013; Blurton Jones, 2016). For the purpose of this study, we visited the Hadza camps and recruited our sample close to the camps they inhabit. The study was performed in the same place as some of our previous psychological and anthropological research on the Hadza (i.e., Butovskaya et al., 2017; Sorokowski et al., 2017a; Groyecka et al., 2019). Due to low literacy rates, participants gave their informed oral consent prior to the study inclusion. Polish participants were recruited in a provincial town in South-West Poland during educational courses at the local University. After agreeing to take part in the study, Polish participants provided a written informed consent.

#### Procedure

The data were collected in August 2017. Data from residents and visitors of seven Hadza camps, located in rural north Tanzania around Lake Eyasi were collected, using a snowball sampling procedure. Participants responded orally to interviewers’ questions during individual sessions. They were assured they are free to resign at any time without consequences. Participants were asked in Swahili to indicate how they felt during the previous week using a 3-point Likert-type scale, where: (1) indicates that they were sad, (2) sometimes happy and sometimes sad, and (3) happy. The questions were simplified as compared to the original scale to provide a better understanding of the happiness term. The same procedure was applied in Poland.

#### Statistical Analyses

Analyses were performed with IBM SPSS v.25 and R packages with the level of significance set to 5%. To examine the factors underlying the quality of life we regressed sex (dummy coded: 0 = male; 1 = female), age (centered around mean), group (contrast coded: −1 = Hadza; 1 = Poland), and an interaction factor *Age x Group* on the happiness. Given the ordinal scale we have used, for multivariate analyses we recoded the answers into a dichotomous variable, by collapsing answers 0 and 1 into 0, and answer 2 into 1. Thus, in this new variable 1 described participants who felt happy. We applied logistic regression to explain the variability in this scale.

### Results

Regression coefficients of the model testing happiness are presented in Table 1. The only significant predictor was group wherein Hadza presented higher happiness (*M* = 2.91) than Polish subjects (*M* = 2.18). Sex and age were not significant predictors. The difference between societies was responsible for 35.7% of the variance in the level of happiness (Nagelkerke *R*^2^).

**TABLE 1:** Regression coefficients for the model predicting happiness from group, sex, age, and age × group interaction.

Predictor	*B*	*SE*	*Exp(B)*	*p*
Sex	–0.06	0.34	0.94	0.858
Age	0.003	0.016	1.003	0.839
Society	–1.446	0.23	0.24	<0.001
*Age* ×*Society*	0.000	0.016	1	0.981

**FIGURE 1:**
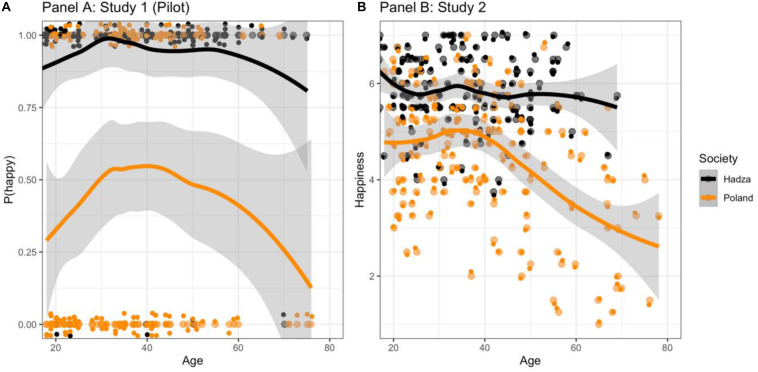
The relationship between happiness and age across Hadza and Poles in Study 1 **(A)** and Study 2 **(B)**.

Apart from a lack of significant interaction effects, in an attempt to explore possible curvilinear links, we exploratorily plotted the relationships between countries looking for possible more complex links. As illustrated in Figure 1A, while in the case of Hadza there were no clear links between age and the likelihood of being happy, among Poles the relationship appeared to be curvilinear. Indeed, when we tested if the curvilinear links observed in Poland were significant, it became apparent that although at first there was no link between age and the likelihood of being happy (β = 0.13, *p* = 0.22), then, the decrease was significant (β = −0.22, *p* = 0.034).

### Short Discussion

Findings from the first study provide evidence that Hadza exhibit higher levels of happiness than Poles. The research task of assessing own’s happiness on a single-item scale turned out to be engaging, and easily comprehended by the Hadza participants. Thus, we decided to further explore the happiness construct in both societies, by using a more sophisticated and established measurement – Subjective Happiness Scale (Lyubomirsky and Lepper, 1999), and not the one-item scale that had not been previously used. Moreover, given the exploratory character of the finding that in Hadza there were no links between age and happiness, while among Poles the relationship appeared to be curvilinear, we attempted to replicate it in Study 2.

## Study 2

### Materials and Methods

#### Participants

Study 2 had the same design, therefore we used the same benchmarks as indicated in the power analysis for Study 1. One-hundred forty-five Hadza (65 females, *M*_age_ = 35,4; SD = 12,5; Md = 34; Min = 16; Max = 69) were recruited for the study and 156 Polish participants constituted a comparison sample (82 females, *M*_age_ = 39,4; *SD* = 15; Md = 37; Min = 18; Max = 78 matched in terms of sex, χ^2^(1) = 1.8, *p* = 0.18, but a little older, *t*(299) = 2.49, *p* = 0.01), but the effect size of this difference was negligible, Cohen’s *d* = 0.29.

#### Procedure

This study was conducted in June 2018. For the purpose of this study, we visited nine Hadza camps, located in rural, north Tanzania around Lake Eyasi. Participants were recruited using a snowball sampling procedure. Due to low literacy rates, participants gave their informed oral consent prior to the study inclusion. Polish participants were recruited in a provincial town in South-West Poland, and provided written informed consent.

Participants responded orally to interviewers’ questions in individual sessions. They were assured they are free to resign at any time without consequences. Participants were asked to answer four simple question from Subjective Happiness Scale (Lyubomirsky and Lepper, 1999), translated into Swahili by the collaborating interpreter. Because of a good comprehension of a Likert-type scale employed in Study 1, we decided to use an original 7-point Likert-type scale from the original version. The final score represents mean of the four scale items. The same procedure was applied in Poland.

#### Statistical Analyses

We started our analyses by testing the cross-cultural equivalence of the happiness scale. To this end, in a multi-group factor analysis in R (package lavaan, see Rosseel, 2012) we compared the models assuming the same one-factor structure across societies (configural invariance), with a model with factor loadings constrained to be equal (metric invariance) and intercepts to be the same in both groups (scalar invariance). When evaluating the model fit, we relied on the usually applied criteria (Hu and Bentler, 1999), in which a comparative fit index (CFI) and Tucker Lewis Index (TLI) above 0.90 indicate adequate fit, whereas a root mean square error of approximation (RMSEA) below 0.08 and a standardized root mean square residual (SRMR) below 0.06 indicates no misfit. However, given that it is well established that RMSEA tends to be biased in studies conducted on small samples (Chen et al., 2008), we relied on CFI/TLI and SRMR instead. When evaluating measurement equivalence, we compared the configural invariance model with metric invariance model, and then metric invariance model with scalar invariance model. As these models were characterized by a growing complexity (each subsequent model was nested within the previous one), while assessing models’ superiority we relied on cut-off criteria recommended for testing measurement invariance (Cheung and Rensvold, 2002; Chen, 2007). More specifically, a change of CFI (Δ*CFI*) of less than 0.01 (Δ*CFI* < 0.01), a change of *RMSEA* of less than 0.015 (Δ*RMSEA* < 0.015), and a change of *SRMR* of less than 0.01 (Δ*SRMR* < 0.01) would indicate that two compared models do not differ in terms of model fit.

Then, using Hayes (2017) process macro (model 1), we tested two models. Model 1 tested if country (society) moderates the link between age and happiness when sex is controlled for. Model 2 tested the same question. Following the unexpected finding from Study 1, we aimed to test possible curvilinear links between age and happiness in Poland, so we examined if the possible links might be curvilinear rather than linear.

### Results

The overall model fit the data well (*CFI* = 0.981, *TLI* = 0.944, *SRMR* = 0.033, *RMSEA* = 0.135, 95% *CI*:0.072,0.21), although, as predicted, the *RMSEA* indicated a misfit, probably caused by a relatively small sample size. Thus, we relied on changes in *CFI* and *TLI* (Δ*CFI* and Δ*TLI*), as well as changes in *SRMR* (Δ*SRMR*) in evaluating measurement invariance.

The configural variance model fit was acceptable (*CFI* = 0.977, *TLI* = 0.931, *SRMR* = 0.035), similarly as the metric invariance model: *CFI* = 0.963, *TLI* = 0.937, *SRMR* = 0.057, while the scalar invariance model was below usually recommended cut-offs: *CFI* = 0.91, *TLI* = 0.89, *SRMR* = 0.10. However, the difference in model fit indices between models indicated that – using proposed criteria – neither metric, nor scalar invariance could be established. Thus, based on recommendations indicating that partial invariance may allow for reasonable comparisons (see e.g., Byrne et al., 1989), we have also estimated models with partial metric and partial scalar invariance (see the R script in the Supplementary Material). As illustrated in Table 2, allowing the loading of item 3 to vary between countries and the intercept of item 2 to be freely estimated significantly improved the model fit, thus indicating that both partial metric and partial scalar invariance are established.

**TABLE 2:** Measurement invariance of the Subjective Happiness Scale.

Model	*CFI*	*TLI*	*SRMR*	Δ *CFI*	Δ*SRMR*	Invariance Established?
Model 1: Configural invariance	0.977	0.931	0.035	–	–	Yes
Model 2: Metric invariance	0.963	0.937	0.057	2 vs. 1, Δ = 0.014	2 vs. 1, Δ = −0.022	No
Model 2a: Partial metric invariance	0.980	0.963	0.035	2a vs. 1, Δ = −0.003	2a vs. 1, Δ = 0	Yes
Model 3: Scalar invariance	0.910	0.893	0.102	3 vs. 2a, Δ = 0.07	3 vs. 2a, Δ = −0.067	No
Model 3a: Partial scalar invariance	0.980	0.960	0.035	3a vs. 2a, Δ = 0	3a vs. 2a, Δ = −0.067	Yes

Having established partial metric and scalar invariance, we proceeded with a moderated regression to examine, if the society moderates the link between age and happiness. Given the pattern discovered in the first study, we tested two models. Model 1 examined linear effects, while Model 2 tested for a curvilinear relationship between age and happiness. As illustrated in Table 3, both models explained a large portion of happiness variability – 39% in the case of Model 1 and 41% in the case of model 2. Both models have shown a large difference between societies in terms of happiness (Model 1: *B* = −1.28, Model 2: *B* = −1.26 – an equivalent of Cohen’s *d* = 1.25), significant, negative effect of age, as well as the expected moderation *Age x Society*. Indeed, when compared across countries, there was no relationship between Age and happiness among Hadza (*r* = −0.08, *p* = 0.34), while a robust negative correlation was found among Poles (*r* = −0.44, *p* < 0.001) – and this difference was statistically significant (Fisher’s *z* = 3.37, *p* < 0.001).

**TABLE 3:** Two regression models explaining cross-country differences in subjective happiness.

	Model 1	Model 2
	
Predictors	*B* (*SE)*	*B* (*SE*)
Society	−1.28 (0.12)***	−1.26 (0.12)***
Sex	0.02 (0.12)	0.03 (0.12)
Age	−0.02 (0.004)***	0.03 (0.02)
*Age* × *Society*	−0.03 (0.001)***	–
Age^2^	–	−0.001 (0.0003)*
*Age^2^* × *Society*	–	−0.0003 (0.0001)**
Model *R*^2^	0.39***	0.41***
Interaction *R*^2^	0.03***	0.02**

*p < 0.05, **p < 0.01, ***p < 0.001.

Model 2, testing a more complex relationship, indicated that when controlling for possibly curvilinear effects of age, there was no statistically significant relationship between age and happiness up to a certain point (about 40 years of age), but after then the happiness tended to decline, while this decline was observed only among Polish participants (see Figure 1B), thus replicating the pattern observed in Study 1.

To assess if the result of higher happiness in Hadza were limited only to comparison with Poles, we further assessed the significance of difference in happiness between other countries, for which means were available in the literature. A summary of levels of happiness in twelve other populations (that used, similarly as in the present study, Subjective Happiness Scale) provide evidence that Hadza may present significantly higher subjective happiness (see Table 4). Nevertheless, what is worth stressing is that the overview analysis includes studies conducted over the period of almost 20 years (1999–2018), and even though all the samples derive from industrialized populations, the levels of happiness are known to change from one year to another (Sachs et al., 2018), what contaminates the results and does not allow for drawing general conclusions.

**TABLE 4:** Comparison between level of happiness in Hadza (*M* = 5.83, *SD* = 0.76) and scores from 12 other populations by the means of one-sample *t*-test.

Country	Participants	Mean age	N	M	SD	References	Difference with Hadza (One-sample *t*-test)	Cohen’s *d*
Russia	Adult community sample (City community)	NA	63	**4.02**	0.93	Lyubomirsky and Lepper, 1999	<0.0001	2.40
Malaysia	Community-based participants recruited from Kuala Lumpur; mean age of the Chinese participants: 33.60 (*SD* = 12.13); of the Malay participants: 32.16 (*SD* = 12.51)	NA	517	**4.42**	1.48	Swami, 2008	<0.0001	1.87
Turkey	Community Sample (Non-students)	39.03	222	**4.73**	1.39	Doğan and Totan, 2013	<0.0001	1.46
Italy	The participants from five regions in the north, the center and the south of Italy (Community Sample); participants age range: 18–85	NA	993	**4.77**	1.22	Iani et al., 2014	<0.0001	1.40
Slovakia	Community samples in Prešov (a medium-sized city in the eastern part of Slovakia); age ranged from 21.58 to 37.86 in seven samples	29.47	270	**4.78**	1.07	Babinčák, 2018	<0.0001	1.39
Philippines	The participants from a community sample in Manila	29.51	182	**4.85**	0.84	Swami et al., 2009	<0.0001	1.30
Chile	People from the general population (city Santiago de Chile)	25.41	300	**5.04**	1.70	Vera-Villarroel et al., 2011	<0.0001	1.05
Hong Kong	People in households in the Hong Kong (The sociodemographic characteristics of the study sample were similar with those of the general population of Hong Kong)	49.2	2635	**5.07**	1.05	Nan et al., 2014	<0.0001	1.01
Spain	Adult participants recruited from the community (Non-students)	48.93	261	**5.12**	1.03	Extremera and Fernández-Berrocal, 2014	<0.0001	0.94
Austria	The participants from a community sample in Vienna and its environs	29.12	960	**5.18**	1.06	Swami et al., 2009	<0.0001	0.86
United States	Adult community sample (City community)	NA	198	**5.62**	0.96	Lyubomirsky and Lepper, 1999	0.01	0.28
Mexico	The sample consisted in the general population (city of Monterrey)	33.14	849	**5.68**	1.04	Quezada et al., 2016	0.047	0.20

## Discussion

The current investigation aimed to explore happiness of Hadza hunter-gatherers and Poles. Results of both studies showed that Hadza reported a higher level of happiness with their lives compared with Polish people. In both studies, sex was not related to participants’ happiness. Exploratory analysis from the first study provided evidence for a curvilinear link between age and happiness among Poles (but not among Hadza). We have then confirmed this hypothesis in the second study, using a validated measurement – Subjective Happiness Scale. Age was a negative predictor of happiness in Poland, whereas no such relationship was observed in Hadza. Therefore, we hypothesize that positive perception of aging in societies may increase their actual happiness (Levy et al., 2002, 2014; Ingrand et al., 2018).

Albeit simpler and more nature-dependent life, highly egalitarian social structure, and a high degree of cooperation Hadza are known for Marlowe (2004, 2009), Apicella et al. (2012), Henrich (2012), can be named among possible reasons to explain the current findings in differences in happiness. Such social organization in Hadza might explain why the level of subjective happiness is independent from age, as opposed to Polish society, wherein subjective happiness declines with age. Perhaps Hadza of old age find their place in the social organization, and their contribution to the society remains high despite not being as fit as in younger age. The best illustration are Hadza grandmothers, investing actively in grandchildren, and providing better chances for their survival (Hawkes et al., 1997; Crittenden and Marlowe, 2008, 2013). Nevertheless, what is worth highlighting is that also numerous other factors can account for the observed differences between Hadza and members of the Western countries, for instance, differences in pollution, access to firearms, or living in communities of millions instead of 20–30 people (Welsch, 2006; Lankford, 2016; Okulicz-Kozaryn and Mazelis, 2018). Moreover, our results are contradictory to findings of previous studies, which suggested that the relationship between age and happiness in more developed, Western societies is U-shaped, with older people showing greater happiness than middle-aged (Blanchflower and Oswald, 2008; Wolpert, 2010). On the other hand, Frijters and Beatton (2012) provided evidence that the widely reported U-shape is just an artifact of the bias of coefficients of variables, which peak in middle-age (e.g., income, marriage, and employment).

Our results are intriguing in the light of previous findings on the material resources being a necessary condition for raising the level of happiness in the population (Devoto et al., 2012; Mahasuweerachai and Pangjai, 2018). Hadza present very remote and mobile way of living. Individuals change camps every few months and the whole population resides where natural resources (e.g., game, plants) allow their subsistence (Woodburn, 2017a, b). The lack of accumulated goods further eases mobility. We speculate that the relatively high level of Hadza happiness is rooted in their social organization and culture directed toward the community. In fact, happiness is related with communal, interpersonally-oriented traits and high positive affect (Furr, 2005). Moreover, many studies highlight the role of social support and social ties in developing and maintaining high levels of happiness and well-being (Chan and Lee, 2006; Aknin et al., 2011). It is, thus, not surprising that positive psychologists, psychotherapists, and even World Bank recommend fostering quality social ties, as they have a significant and positive effect on happiness (World Development Report, 2003; Lakey, 2013; Compton and Hoffman, 2019). Although evidence for the relationship between the social support, communion and happiness comes from the modern societies (Hermans, 1992; Helgeson, 1994), we speculate that communal behaviors are present and promoted among Hadza to a larger extent, as they manifests in hunting, sharing food and motherhood challenges (Hawkes et al., 1989, 2001; Marlowe, 2009; Apicella et al., 2012; Henrich, 2012), which can translate into a high level of happiness.

In the present research, we have used two measures of happiness – in the first study, we assessed happiness by referring to an affective aspect, whereas in the second study, we used Subjective Happiness Scale, which relates both to the affective and cognitive components (Lyubomirsky and Lepper, 1999). This may be important, as there are various definitions of happiness, including ones based entirely on emotions, and others based purely on thinking (Veenhoven, 2009). Findings of the present study suggest that both cognitively and affectively perceived happiness is higher among Hadza in comparison with more industrialized societies.

It would be interesting to test whether hunter-gatherer societies have higher happiness than pastoralists and agriculturalists. Agriculture opens the possibility for social stratification and exploitation. That is why some authors suggested that from the viewpoint of individual happiness, the “agricultural revolution” was the worst mistake in the history of the human race (see Harari, 2014). Such hypothesis could be explored in Hadza, whose territories are surrounded by Datoga pastoralists, and Iraqw agro-pastoralists. Moreover, as happiness can be affected by numerous factors (e.g., standard of living, or relationships), future studies should focus on exploring possible moderators of happiness in more traditional populations.

The readers may wonder whether the Hadza fully comprehended the given task (i.e., rating their happiness level). The authors have years of experience in conducting such studies, especially among the Hadza population, thus, with fairly high certainty, we can ensure that our participants understood the scales used in the present study. When the Hadza do not understand the question they are being asked, not only their facial expressions and non-verbal behavior change, but they also admit they do not comprehend the given task. Usually, when we have concerns about the question comprehension, we randomly ask the same questions twice. As we had no doubts that the Hadza understood the task of the present study, we did not perform such checking techniques. Nevertheless, this can be regarded as a potential limitation of the present study (and in general, many other studies conducted among the traditional, illiterate populations).

Another possible limitation of the present findings is that, due to the small samples, recruiting techniques, and lack of extensive information regarding participants from both populations, final samples do not necessarily represent well the whole societies they derive from (i.e., Polish and Hadza). Similarly, we chose Hadza and Poles as representatives of traditional and modern societies, having no certainty that Poles are indeed typical for all non-Western countries and Hadza do not necessarily represent all hunter-gatherer cultures. Nevertheless, Poles do present a modern, industrialized way of living and as members of European Union are strongly bonded with and influenced by other cultures of the West. Yet, one needs to bear these in mind when interpreting the results of the present study.

We would also like to note the fact that some of our statistical decisions may be considered suboptimal. In the first study we merged two categories of answers, namely “sad” and “sometimes happy and sometimes sad” into one category. We acknowledge that this decision might be considered problematic, but because we were interested in “happiness,” it made sense to dichotomize our participants into those who considered themselves “happy” vs. the rest. In the second study, we did not test for invariance in each group (i.e., Poles and Hadza) separately. Instead, we tested configural, metric and scalar invariance in multi-group CFA: a solution that resulted in higher statistical power of our analyses.

## Data Availability Statement

All datasets generated for this study are fully available without restriction in Supplementary Material.

## Ethics Statement

This study was carried out in accordance with project NIR No. 01201370995 “Cross-cultural and interdisciplinary researches. Biosocial and cross-cultural analysis of models of tolerance and basic values of culture in modern society,” and approved by Commission for Science and Technology of Tanzania and by the Institutional Review Board (IRB) of the University of Wrocław (Wrocław, Poland) with written informed consent from all subjects. All participants gave informed consent in accordance with the Declaration of Helsinki. Hadza participants are illiterate – gave their informed oral consent prior to the study procedure. Polish participants provided a written informed consent. Both consent procedures were approved by the Commission for Science and Technology of Tanzania and by the Institutional Review Board (IRB) of the University of Wrocław (Wrocław, Poland).

## Author Contributions

TF and PS contributed to the conception and design of the study. PS, TF, MB, and AG collected the data. MKa, TF, AO, and PS performed the statistical analysis. TF, AO, AG, MKo, MKa, PS, and MB wrote the first draft of the manuscript. PS and MKo contributed to manuscript revision.

## Conflict of Interest

The authors declare that the research was conducted in the absence of any commercial or financial relationships that could be construed as a potential conflict of interest.
