# Supplementary material for: Subjective Happiness Among Polish and Hadza People
Source: Front Psychol. 2020 Jun 9;11:1173. doi: 10.3389/fpsyg.2020.01173 (PMC7296072; doi:10.3389/fpsyg.2020.01173)
Supplement: Supplementary file 2 [file Table_2.DOCX]

setwd(PATH)

data <- read.csv("DATA.csv", sep = ",", dec=".")

model <- 'sat =~q1+q2+q3+q4'

Model 1: Configural invariance model

fit <- cfa(model,

data = data,

group = "Society..1.Hadza.2.Poland.")

summary(fit, fit.measures=TRUE, standardized=TRUE)

Model 2: Metric Invariance model

fit <- cfa(model,

data = data,

group = "Society..1.Hadza.2.Poland.",

group.equal = c("loadings"))

summary(fit, fit.measures=TRUE, standardized=TRUE)

Model 2a: Partial Metric Invariance model

fit <- cfa(model,

data = data,

group = "Society..1.Hadza.2.Poland.",

group.equal = c("loadings"),

group.partial = c("sat=~q3"))

summary(fit, fit.measures=TRUE, standardized=TRUE)

Model 3: Scalar Invariance

fit <- cfa(model,

data = data,

group = "Society..1.Hadza.2.Poland.",

group.equal = c("loadings", "intercepts"))

summary(fit, fit.measures=TRUE, standardized=TRUE)

Model 3a: Partial scalar invariance

fit <- cfa(model,

data = data,

group = "Society..1.Hadza.2.Poland.",

group.equal = c("loadings", "intercepts"),

group.partial = c("sat=~q3", "q1~1", "q2", "q3~1", "q4~1"))

summary(fit, fit.measures=TRUE, standardized
